# Health impact of the Anthropocene: the complex relationship between gut microbiota, epigenetics, and human health, using obesity as an example

**DOI:** 10.1017/gheg.2020.2

**Published:** 2020-04-20

**Authors:** Cecilie Torp Austvoll, Valentina Gallo, Doreen Montag

**Affiliations:** 1 Centre for Primary Care and Public Health, Queen Mary University of London, London, UK; 2 London School of Hygiene and Tropical Medicine, London, UK; 3 School of Public Health, Imperial College London, London, UK

**Keywords:** Anthropocene, biodiversity loss, epigenetics, global health, microbiota, obesity

## Abstract

The growing prevalence of obesity worldwide poses a public health challenge in the current geological epoch, the Anthropocene. Global changes caused by urbanisation, loss of biodiversity, industrialisation, and land-use are happening alongside microbiota dysbiosis and increasing obesity prevalence. How alterations of the gut microbiota are associated with obesity and the epigenetic mechanism mediating this and other health outcome associations are in the process of being unveiled. Epigenetics is emerging as a key mechanism mediating the interaction between human body and the environment in producing disease. Evidence suggests that the gut microbiota plays a role in obesity as it contributes to different mechanisms, such as metabolism, body weight and composition, inflammatory responses, insulin signalling, and energy extraction from food. Consistently, obese people tend to have a different epigenetic profile compared to non-obese. However, evidence is usually scattered and there is a growing need for a structured framework to conceptualise this complexity and to help shaping complex solutions. In this paper, we propose a framework to analyse the observed associations between the alterations of microbiota and health outcomes and the role of epigenetic mechanisms underlying them using obesity as an example, in the current context of global changes within the Anthropocene.

## Introduction

**Fig. 1.**
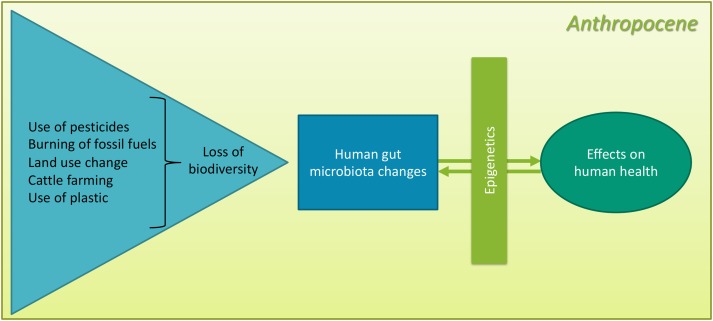
Framework analysing the health effects of loss of biodiversity in the Anthropocene.

In this paper, we will analyse the Anthropocene as the context in which human actions are continuously leading to global change that is resulting in mass-extinction and biodiversity loss. The anthropogenic planetary context is defining humans' experiences of health and well-being, their relationships with the environment, risks to and experiences of ill-health and diseases [1]. Biodiversity loss has a direct impact on human health [2]. One of the pathways of impact is related to the microbiota. Biodiversity loss is directly impacting the microbiota diversity of humans, soil and other species, which are interrelated [3]. Decreased diversity of the human gut microbiota during the development phase and during later life course can have several impacts on health outcomes [4,5]. One of the pathways of interaction between the human gut microbiota and health outcomes is through epigenetics. This can be exemplified through the current obesity epidemic. A framework capturing the complex interaction between the anthropogenic activities and their impact on health through the reduction of biodiversity and epigenetic changes has been constructed (Fig. 1).

In this paper, the existing scientific evidence will be reviewed and analysed within this proposed framework, using obesity as an example. This paper addresses the growing interest in microbiota in relation to health that seems to be (partly) mediated via epigenetics. The paper gives an overview over existing data, providing advice for future research and public health directions on this topic.

## Anthropocene

The Anthropocene is the new geological epoch where anthropogenic activities, such as the burning of fossil fuels (technology and infrastructure) and land use change (agriculture and urbanisation), are shaping and have led to a dysbiosis in planetary processes [6, 7]. Anthropogenic activities led to a global change, including increased use of pesticides, use of plastics (a derivate of oil) and other contaminants that are polluting oceans, air and soil, leading to changes at the planetary scale [8].

Planetary changes include climate change and biodiversity loss [8]. Climate change has a direct impact on biodiversity, which, in turn, is impacting climate change through its central role in ecosystem health, regulating local and regional climate [9–13]. Deteriorated local, regional and planetary ecosystems play a central role in influencing population health, putting people at higher risk for a range of infectious and non-communicable diseases, such as obesity, which have gained more momentum in research since the WHO Ecosystem Millennium Assessment in 2005 [14], the WHO/CBD State of Knowledge Review on Biodiversity and Human Health [15] and particularly since Whitmee *et al*. [1] defining work on Planetary Health.

Biodiversity and genetic (intraspecies) and species loss are direct consequences of the global change in characterising the Anthropocene [13, 16]. It is impacting food security, microbial ecology and functionality, and, above all, human health [15]. Microbial ecology and functionality play a central role in the human microbiota gut, through interaction with environmental microbial diversity in soil and food over a life-span [7, 17–22]. Humans have evolved within the planetary system and are dependent upon its functioning local, regional and planetary ecosystems. Human gut microbiota and immune system have co-evolved due to exposure to various microbes in the surrounding environment [23], such as helminths; or, as Rook [24] defines them, as ‘immunoregulatory old friends’ which have been lost through global changes, biodiversity loss in the soil environment [25–27].

The impact of global change and biodiversity loss in the context of the Anthropocene on the human gut microbiota has not been directly analysed yet. While the Anthropocene can be defined as a dysbiosis of the planetary system, a dysbiosis of the human gut microbiota could be seen as a resemblance of this on an ecosystem level, indicating a systemic dysbiosis on the micro and macro levels of the planetary system.

## The human gut microbiota

The terms microbiota and microbiome are often used interchangeably. In this paper, the term microbiota is used to refer to all microorganisms that reside within the human body, and the term microbiome to their genomes and genetic information [28]. The average ratio of bacteria cells to human cells has been estimated to be 1:1 [29–31]. Most of the bacteria are located in the large intestine and on the human skin, with *Bacteroidetes* and *Firmicutes*, as main phyla accounting for >90% of the total gut microbiota [29].

The human microbiota gut is formed of phyla, bacterial species and strains, yeasts and other microbes [32]. It is important for maintaining human health, playing a role in proper digestion, synthesis of vitamins, production of neurotransmitters, absorption of minerals, regulating the immune system and inflammatory response while preserving the integrity of the gut epithelial barrier [33–37].

The development of the human gut microbiota composition in the first 2 years of life defines the immune system among other functions, central for child development and growth [28]. Several studies have found an association with lower diversity in the gut and chronic inflammation, thereby influencing obesity and other non communicable diseases (NCDs), such as allergies, diabetes, cancer and some psychiatric disorders [16, 24–27, 38–47].

## The effects of the Anthropocene on the human gut microbiota

A recent review of geographical differences in gut microbiota with diet showed that people eating an omnivorous diet had a higher diversity of bacteria compared to vegetarians [48]. Moreover, gut microbiota composition differs widely according to a geographical area and between different ethnic groups within the same area, with the highest diversity of bacteria species encountered in the African population [48]. A comparative study of gut microbiota among Colombians, Europeans and Asians found that in Colombians, there is a tendency in *Firmicutes* diminishing with increasing body mass index (BMI), whereas no association was observed for *Bacteroidetes* [49]. Escobar et al. [49] pointed out that geography contributed to determining bacteria composition more than BMI or gender.

Research by McDade *et al*. [50] in a rural Ecuadorian Amazonian context found no existing chronic background inflammation among residents. Blackwell *et al*. [51] reported similar results among Bolivian Amazonian foraging horticulturalists with higher inflammatory indicators in younger age which are depleted in later years of life. Further research has shown that babies that have been exposed to unharmful infections (old friends) in early childhood have a stronger immune system and low chronic inflammation in later life [52, 53]. Similar results have been reported from other Ecuadorian Amazonian and Peruvian Amazonian contexts [54, 55]. Chronic background inflammation is directly related to metabolic disorders, of which obesity is one.

Recent studies on the diverse human gut microbial functionality have looked at the impact of ‘westernisation’ and industrialisation; how ‘cultural change’ have impacted human gut microbiota by looking at hunter-gather groups, people living in rural and urban contexts [56, 57]. Clemente *et al*. [56] analysed faeces, skin and oral samples among rural Yanomami people in the Venezuelan Amazon region. They demonstrated an even more diverse composition and with the lowest variability of human microbiota than those of ‘semitransculturated’ Guahibo Amerindians and Malawians. The microbiome was similar across Yanomami people than across other study participants. Clemente *et al*. [56] concluded that the way of living, having been isolated in the Amazon in contrast to a ‘semi-westernised’ lifestyle had an essential impact on the microbiota composition. Yatsunenko *et al*. [57] conducted a cohort study among Venezuelan Amazonian, rural Malawian and urban US people on the impact of microbiota between age and geography. They found a difference in ‘bacterial assemblage and functional gene repertoires’ (p. 222) between the first two more rural Venezuelan and Malawian and the urban US populations with similarities across age [57]. They concluded that a difference in the diet has contributed to the distinct adult microbiota. Diet then is associated with lifestyle and social structure [57]. In another study on seasonality and food consumption and impact on human gut microbiota among Hadza hunter-gatherers in Tanzania, Smits *et al*. [58] have demonstrated that seasonality and availability of food and food quality plays a role in the human gut microbiota among people with a very biodiverse and a highly functional human gut microbiota. Smits *et al*. [58] presented that while Firmicutes composition was the same during different seasons, Bacteroidetes operational taxonomic units changed. In comparison with 18 different populations from 16 distinct countries, they conclude that those from more agricultural and rural hunter-gatherer areas where higher in Prevotellaceae than those from urbanised and industrialised contexts. Commonalities were also found with the existence of Spirochaetaceae and Succinivibrionaceae among agricultural and rural areas, and the seasonal disappearance of Bacteroidetes taxa was shown similar to those generally encountered among people living in industrialised contexts [58]. They concluded that there is a substantial ‘cultural’ difference between human gut microbiota [58]. This evidence suggests that the analysis of the association between gut microbiota and obesity must be geographical location dependent, and the comparison between distant geographical locations would be invaluable in unveiling underlying mechanisms.

## Epigenetics and epigenetic pathways

Epigenetics is the study of heritable changes which affect gene functioning without modifying the DNA sequence [59, 60]. Epigenetic patterns are shaped dynamically throughout the life-course, and vary from cell types, in contrast to the genetic sequence. The ways epigenetic changes regulate DNA expression and cell maintenance are mainly attributed to the covalent modification of DNA by methylation [61].

Epigenetic mechanisms have been associated with the microbiota in their modulation of weight, metabolism, appetite control, insulin signalling and inflammation through metabolite production [62–67]. These mechanisms are gaining progressively more attention as potentially explaining the growing prevalence of obesity worldwide [34, 68].

There is evidence to show that epigenetics plays a vital role in transmitting obesity and type-2 diabetes risk to the offspring [69]. Current research has also shown that obese people tend to have different epigenetic patterns compared to non-obese, reinforcing the relative importance of epigenetics in the study of obesity [70–73].

## The role of the gut microbiota in human health using obesity as an example

The development of the early human gut microbiota and immune system and future influences through food intake are essential when approaching obesity. The modulation of host energy balance (intake and type of food, food behaviour, intestinal absorption, energy recovery from the diet and the anabolic/catabolic balance) and others have concluded that obesity can be viewed as a condition of persistent low-grade inflammation and inflammatory disease [74–78].

The obesity epidemic has become a primary global public health concern as the prevalence of obesity has been growing fast and steady since the 1970s, but at different rates across nations [39]. According to the most comprehensive analysis, by 2025, the global obesity prevalence will reach 18% in men and 21% in women, while severe obesity will reach 6% in men and 9% in women [79]. Within the global burden of obesity, global childhood obesity has risen dramatically over the last few decades: children are increasingly becoming heavier worldwide [80] and obese children are at higher risk of becoming obese and overweight adults [68].

Obesity is defined by an excessive accumulation of fat mass within the body [81]. According to the thrifty genotype hypothesis [82], the current human predisposition to fat accumulation is the result of an evolutionary selection of people with specific genetic combinations which have made them more resistant to the hunger/feast diet. This same genetic predisposition, in a modern obesogenic environment with constant access to food alongside urbanisation and sedentary lifestyles, has generated a higher prevalence of obesity and overweight [83]. There is also a link between mitochondrial abnormalities and metabolic disorders, such as obesity, diabetes and insulin resistance, suggesting that excessive energy stores have adverse effects on lipid and glucose metabolism, as it may decrease insulin sensitivity within muscle, liver and adipose tissue and thereby disrupting the balance between energy storage and expenditure [84–86]. Obesity has increased alongside the establishment of modern developed states, social welfare systems and economic structures [39, 87–89]. Current projections estimate a shifting burden of obesity towards the poorer and lower-income nations, as many of them are dramatically changing their diets towards high energy-dense foods often lacking essential nutrients [83].

Some genetic determinants play a role in the development of obesity; monogenic forms of severe early onset obesity in children have been described, such as Biedl syndrome or Prader–Willi syndrome [90]. The primary mechanism which has been suggested to explain – at least partially – these associations is an epigenetic modification of DNA expression [91]. The ways epigenetic changes regulate DNA expression and cell maintenance are mainly attributed to the covalent modification of DNA by methylation [91]. Current research has also shown that obese people tend to have different epigenetic patterns compared to non-obese, reinforcing the relative importance of epigenetics in the study of obesity [70–73, 92].

In humans, the microbiota composition is usually different in lean and obese people with obese having showed a reduction in Bacteroidetes accompanied by a rise in Firmicutes [34, 36, 62, 63, 66, 68, 93, 94]. Evidence shows that some bacteria, particular in the Firmicutes phyla, are better at harvesting energy from the food than other phyla and bacterial species thereby contributing to weight gain [34, 65, 68, 93]. Remely *et al*. [94] also found a significantly higher ratio of Firmicutes and Bacteroidetes in type-2 diabetics compared to lean controls and obese. Others have shown no difference between the two phyla in obese and lean controls [29, 34, 93], hence illustrating how a rise in phyla may indicate different results in different people or might be a consequence of status rather than a cause. Also, in the phyla of Firmicutes, there are both so-called beneficial bacteria and Gram negatives; hence, more research is needed to see what types of bacteria, strains and species within the phyla that are in particular linked to excess body weight or linked to changes in how bacteria extract energy from the diet.

A lack of diversity in the microbiota has been associated with dysbiosis in the gut and low-grade chronic inflammation that promotes metabolic disorders, such as obesity and type-2 diabetes in both humans and animals [34, 64, 94–96]. Importantly, the ecosystem of the microbiota continues to change throughout a life course and is likely to be affected by epigenetics [97]. Following, the microbiota is becoming increasingly more recognised as an influencer in epigenetic modifications that takes place throughout a life course [68]. With this, more research needs to be done in order to fully comprehend the relationship between epigenetics and obesity, in terms of what is the first modulator.

Epigenetic mechanisms have been associated with the microbiota in their modulation of weight, metabolism, appetite control, insulin signalling and inflammation through metabolite production [62–67]. These mechanisms are gaining progressively more attention as potentially explaining the growing prevalence of obesity worldwide [34, 68].

The combination of potential genetic/epigenetic, social and environmental risk factors for obesity, has prompted research to focus on the variation of individual risk within obesogenic environments; e.g. epigenetic processes that take place in early life, energy-rich environments such as infant over-nutrition, and maternal obesity, which can significantly increase the risk of obesity later in life [91]. This has contributed to a shift towards epigenetic mechanisms, and to how genes are regulated and expressed throughout a life course [98]. Nevertheless, epigenetic changes and obesity outcomes should be considered into a broader approach accounting for the complexity of the issue, new developments of understanding of the gut microbiota concerning biodiversity in surrounding environments and the importance of the gut microbiota in the context of the Anthropocene [25, 27, 73, 99].

## Early life factors

Some research has emphasised the importance of preserving the microbial ecology of the gastrointestinal tract during early development, i.e. pre-natal, in pregnant women and foetuses after birth. The microbiota development is expected to begin at birth when babies pass through the vaginal canal where they are exposed to the mother's bacteria and also through breastfeeding [68]. New research has also indicated that the colonisation of microbes may begin even before birth, as some live bacteria get transferred across the placenta hence indicating the importance of nurturing the gut during pre-natal and during pregnancy [100].

It is estimated that humans establish their full microbiota within the first 2–3 years of life [28, 36, 66]. Increasing importance has been given to ‘windows of opportunity’ for preventing obesity and other metabolic disorders in early life. This might include proper nutrition during pregnancy and breastfeeding and avoiding antibiotics and caesarean section (C-section) whenever possible [28, 101–103]. Caesarean delivery has been associated with increased body mass in childhood and adolescence [104] and with an increased risk of both overweight and obesity in preschool children [105]. Exposure to antibiotics before 6 months of age or during infancy has been associated with increased body mass in healthy children [106]; and evidence suggests that antibiotics may permanently dysregulate foetal metabolic patterns as they can alter epigenetic pathways or maternal microbiota [106, 107]. The offspring of malnourished parents (either over- or under-nourished) have an increased risk of developing both diabetes 1 and 2 and obesity as a result of the changes in the gut microbiota and epigenetic markers [66, 108].

## Exposure to antibiotics *in utero* or very early life and risk of obesity

Prenatal exposure to antibiotics was found to be associated with childhood obesity [109, 110]. The association between antibiotic use and obesity was stronger in babies born with a higher birth weight (>3500 g), while the association with overweight was stronger among babies born smaller (≤3500 g) [109]. The association was maintained during all pregnancy period, without meaningful differences [110].

Early infancy exposure to antibiotics was consistently found to be associated with an increased risk of obesity later in life [106, 111, 112]. Cumulative exposure to broad-spectrum antibiotics in early life was found to be associated with an increased risk of obesity [112]. The effect was maintained in exposure at both very early ages (0–5 months) and later (5–11 months). Interestingly, narrow-spectrum antibiotics were not associated with an increased risk of obesity in any of the age groups considered, suggesting that they could not reach or alter the gut microbiota [112]. Consistently, macrolides, a type of broad-spectrum antibiotics were found to be more strongly associated with obesity compared to other molecules [106]. The association between antibiotic use within the first 24 months and obesity was found to be stronger in boys than girls, and with similar cumulative effects [106].

Antibiotics were found to modify the association between maternal and child body weight. In an analysis of the Danish National Birth Cohort, a strong association between maternal the BMI and child BMI at age 7 was found [111]. This could be explained through a different mechanism including genetic/epigenetic factors, social and behavioural, or through the transmission of gut microbiota at the time of delivery. Antibiotic use before age 6 months interacts with this association, increasing the risk of obesity in children born by normal weight mother, but decreasing it in children born by overweight one [111]. These results suggest that gut microbiota transmission might have a predominant role in explaining mother–child concordance for body weight.

## Caesarean section and risk of obesity

Delivery by C-section reduces the ability of the new born to come into contact with the vaginal and faecal microbiota of the mother during birth. Therefore, they miss this physiological source of bacterial colonisation.

Delivery via C-section was consistently associated with an increased risk of obesity later in life [104, 105, 110, 113]. In meta-analysis, children born by C-section were more likely to be obese by the time they reach 5 years [113]. In one of the studies, by age 11, caesarean-delivered children had almost doubled risk of being overweight or obese. This association was stronger and longer lasting among children born from overweight/obese mothers than from normal-weight mothers [104]. This partially contradicts the interaction maternal-child weight with antibiotic use [111]. Risk estimate was similar for delivery by planned or emergency C-section [110]. To what extent C-section has also linked to alterations in the microbiota needs further examination.

## Mode of infant feeding and impact on gut microbiota and obesity

Breastfeeding contributes to the protection against obesity in children [114]. Breastfeeding at 1 month of age and for more than 6 months was associated with the maximum inverse associations, in one study [115]. Gut microbiota and its dysbiosis in very early ages were shown to play a vital role in this association, as infant exclusively breastfed or formula fed had radically different microbes profiles, with partially breastfed infants having an intermediate profile [116]. Interestingly, among partially breastfed infants, formula supplementation was associated with a profile similar to that of non-breastfed infants, whereas the introduction of complementary foods without formula was associated with a profile more similar to that of exclusively breastfed infants [116].

## Factors associated with obesity later in life

Through the life course, many factors have shown to have an impact on the microbiota, such as diet, nutrition, antibiotics, disease, genetics and exposure to medications [29]. Growing evidence also supports the association between human microbiota and obesity and several studies have demonstrated how the ‘indigenous’ gut microbiota plays a crucial role as an epigenetic regulator via epigenetic modifications that impact gene expression at different life stages [68].

There have been studies suggesting that an increase of members of the Firmicutes phylum leads to elevated short-chain fatty acids (SCFAs), such as butyrate, and increased energy extraction from the diet in addition to promoting the maintenance of the intestinal epithelium [68]. The SCFAs have been found to influence the epigenetic regulations of genes in obese subjects and how an epigenetic mechanism in the gut microbiota may be altered due to nutrition [108].

SCFAs are also believed to engage the epigenetic regulation of inflammatory reactions via a free fatty acid receptor (FFAR) and other short-chain fatty acid receptors [94]. They have also been linked to different levels of the satiety hormone, which could lead to an increase in food intake [36]. Besides, these may shape epigenetic mechanisms, and for example, butyrate is known as a potent histone deacetylate inhibitor thereby playing a role in metabolic processes [68]. There is also an association between the microbiota and T-cell differentiation linking gut dysbiosis to changes affecting the Th17/Treg balance under inflammatory digestive conditions and are also relevant in the early stages of obesity and insulin resistance [64].

Another way of modifying the gut microbiota is through diet. As our gut microbiota is very dynamic, it can easily be profoundly affected by external exposures, such as diet, lifestyle, epigenetics, genetics age, nutrition, medication and other environmental factors influencing the diversity of the gut microbiota [117, 118]. In mice, switching from low fat, plant-based diet rich in fibre, to a ‘Western diet’ high in fat and sugar altered the bacteria composition within a single day [45]. In humans, ‘Western’ high-fat diets have resulted in a reduction in Bacteroidetes and an increase in Firmicutes and foods high in fibre have shown to increase the phylum of Bacteroidetes and to a more diverse microbiota [34]. Others have shown that gut dysbiosis can be altered by a diet rich in non-digestible but fermentable carbohydrates, which were found to promote significant weight loss [90].

Several studies have stated that epigenetic processes in relation to the gut microbiota play a crucial position in the development of obesity and other metabolic disorders, as bacteria can cause changes in the DNA methylation patterns of host cells by providing epigenetically active metabolites and substances, and these metabolites are essential for DNA methylation so vital for humans [34, 35, 63–66, 68, 93, 94].

## Effects of diet and/or probiotic supplementation on the alteration in body composition and microbiota

The role of gut microbiota in diet-related obesity and some genetic forms of obesity has been investigated in a clinical trial including children with Prader–Willi syndrome and diet-related obesity [90]. A diet rich in non-digestible carbohydrates induced significant weight loss and concomitant structural changes of the gut microbiota in both groups, together with the alleviation of inflammation. This change was also accompanied by a relative increase of functional genome groups for acetate production from carbohydrates fermentation in the gut. These findings suggest a role of gut dysbiosis in obesity which is independent of the aetiology of the condition [90].

However, not all probiotics impact dysbiosis in the same way. Supplementation with galactooligosaccharides among overweight and obese men and women selectively increased the abundance of *Bifidobacterium* species in faeces by five-fold (*p* = 0.009) [119]. However, this did not contribute to significant changes in insulin sensitivity, as no significant alterations in peripheral and adipose tissue, insulin sensitivity, body composition, energy and substrate metabolism were found [119].

A complex double-blind, randomised cross-over clinical trial was conducted to examine the exposure to probiotics on psychological state, eating behaviour and body composition among women [120]. Study subjects were classified as (1) metabolically obese/normal-weight [121]; (2) metabolically healthy/obese [122]; (3) metabolically unhealthy/obese or ‘at risk’ obese [120] and (4) normal weight obese syndrome [123]. An insufficient, but significant, reduction in BMI, body resistance, fat mass (kg and %) and a substantial increase in free fatty mass (kg and %) were observed in all normal-weight/obese and pre-obese/obese subjects after probiotic intake. In the same groups, a reduction of bacterial overgrowth syndrome and lower psychopathological scores were observed after the intervention [120].

## The role of the gut microbiota composition

A relative abundance of *Akkermansia muciniphila* was shown to be negatively associated with BMI in the animal models of obese mice [124], in pregnant women [125, 126] and overweight children [127]. Interestingly, however, the same alteration was also observed in adults within the normal range of BMI: a stool sample of Korean twins who were either obese or diabetic but included a broad spectrum of phenotypes was analysed to explore the distribution of gut microbiota in relation to body weight [128]. For both clinical and microbial phenotypes, longitudinal samples (samples of the same individual taken over time) were more similar than those of twins; however, the twins were more similar than unrelated individuals. The abundance of *A. muciniphila* was negatively associated with BMI, fasting blood sugar and insulin levels [128].

Some changes in microbiota were shown to be causally related to obesity rather than the other way around, through clinical trials. A randomised, double-blind, placebo-controlled study to evaluate the efficacy of transglucosidase (TGD) in modulating blood glucose levels and body weight gain in patients with type-2 diabetes showed that the *Bacteroidetes-to-Firmicutes* ratio in the TGD groups significantly increased compared to the placebo group after 12 weeks. This, in turn, was associated with decreased blood glucose levels and prevention of body weight gain [129].

## The role of epigenetics in explaining the association between gut microbiota and obesity

The abundance of specific phyla and bacteria in the microbiome in association with epigenetic changes was studied in a pilot study on pregnant women [63]. The association between relative abundances of the predominant phyla in the gut microbiota and whole-genome methylation analysis was studied. DNA methylation patterns in white blood cells were associated with gut microbiota profiles, in particular comparing mothers with higher levels of *Firmicutes* with mothers with higher levels of *Bacteroidetes* and *Proteobacteria*. Pathway analysis revealed potential associations between gut microbiota relative abundance and cardiovascular diseases, inflammatory response, metabolic pathways and cancer.

Data from a Norwegian birth cohort of 552 children were used to sequence 16S rRNA genes on gut microbiota among 169 women, 4 days after delivery and 844 samples of their infants at six-time points during the first 2 years of life [130]. These data were used to measure how pre-pregnancy weight and gestational weight gain influence the gut microbiota of mothers during delivery and of their infants in early life. While maternal gut microbiota was found to vary according to pre-gestational weight and gestational weight change, these were only weakly associated with compositional differences in the gut microbiota of their infants [130].

Similarly, differences between 16S rRNA gene sequencing data across normal BMI, overweight and obese groups were found with diversity decreasing in the obese when compared with the normal group, with or without diet confounding factors, in a cross-sectional study in a Korean population [131].

Finally, a placebo-controlled intervention study to evaluate the effect of supplementation with GLP-1 agonists (glucagon-like peptide-1 agonists) on the bacteria composition in insulin-dependent type-2 diabetic individuals, obese and lean non-diabetic individuals using a methylation analysis was evaluated. In comparison with lean individuals, the abundance of *Faecalibacterium prausnitzii* and microbiota diversity was remarkably lower in obese and type-2 diabetic subjects. The analysis of five CpGs in the promoter region of FFAR3 showed significant lower methylation in obese and type-2 diabetics. It increased in obese patients throughout the period. These results unveiled a substantial correlation between a higher BMI and lower methylation of FFAR3. Conversely, LINE-1, a marker of global methylation, indicated no significant differences between the three groups or the time points, although the methylation of type-2 diabetics tended to increase over time.

## Interactions of the gut microbiota, obesity and epigenetic mechanisms in the Anthropocene

More research has pointed out how our microbiota has geographical characteristics, thereby indicating that the geographic origin and environment also play a role concerning human ecosystems [56–58, 132] and that geography and ethnicity play a role in microbial composition in humans [117]. People living in industrialised societies have shown to have a different bacteria composition and often to be less diverse than non-urbanised and indigenous populations [55, 58]. Moreover, De Filippo *et al*. [133] analysed children from rural places in South-Saharan Africa eating a diet very high in fibre which showed a very different microbiota composition compared to European children, in which the children in Europe were more likely to have a dominance of Firmicutes compared to Bacteroidetes, which is similar to [58]. What this literature had in common was describing the differences based on the so-called ‘culture’ concerning lifestyle, such as ‘westernisation’ and geography, in terms of industrialised, urban, rural and isolated contexts.

Geography in this sense could be seen as an indicator for a functioning ecosystem, disturbed and destructed ecosystem if one looks at isolated Amazonian contexts, rural contexts in Amazonia and Malawi and urban contexts in the USA respectively. Anthropogenic actions altering planetary processes characterise the Anthropocene. Indigenous anthropogenic impact on the Amazon overall biodiversity and soil biodiversity has been demonstrated as increasing biodiversity for 4500 years [134, 135]. Deforestation is decreasing soil biodiversity [136]. None of soil diversity changes has been analysed in any of the studies. However, the consistency of the gut microbiota in humans have been developed and nurtured as a result of human interaction with nature, as in the form of early human settlement during the geographical epoch of the Holocene, with the development of agricultural practices and changes in dietary habits [25, 27]. Rook's research [24–27, 38, 40–43] has been essential to our understanding of the co-evolvement of the human gut microbiota with its environment. The importance of the soil diversity, particularly the existence of specific species ‘old friends’ as Rook points out and their loss during the Anthropocene need to be taken into account when analysing the development of human gut microbiota and geographical differences. Lifestyle seems to be a too simplistic explanation for a more systemic change with planetary consequences.

Moreover, research by Robinson *et al*. [137] is advocating for landscape architecture from a microbiome-ecosystem perspective, which is also supported by a meta-analysis on the positive aspects of gardening on human health [138]. These could then also be analysed from a One Health [139] perspective, including microbiota changes in different species and contexts, with a particular focus on obese cats and dogs [140–143]. Under this circumstance, obesity needs to be analysed in context, and we suggest as a consequence of a global change in the Anthropocene, summing events such as urbanisation, deforestation, transportation, land-use change, changes in agricultural practices, use of pesticides and loss of soil biodiversity [8, 144, 145].

## Conclusion

The role of the gut microbiota, obesity and epigenetic mechanisms is increasingly recognised. Obesity should be understood with environmental variables which are in turn embedded in the current context of global change and particularly biodiversity loss within the Anthropocene. Further research should take into account biodiversity, microbiota and epigenetic changes when developing new obesity research streams. These population-based approached based on a systemic response should complement incentives to combat the growing obesity prevalence at the individual level. All interventions, including systemic, public health response to obesity will need to focus on building intersectional and interdisciplinary strategies that seek to understand the complexity of obesity in the Anthropocene.
